# Recombination hotspots and host susceptibility modulate the adaptive value of recombination during maize streak virus evolution

**DOI:** 10.1186/1471-2148-11-350

**Published:** 2011-12-02

**Authors:** Adérito L Monjane, Eric van der Walt, Arvind Varsani, Edward P Rybicki, Darren P Martin

**Affiliations:** 1 Department of Molecular and Cell Biology, University of Cape Town, Rondebosch, 7701, Cape Town, South Africa; 2 Kapa Biosystems, P.O. Box 12961, Mowbray, 7705, South Africa; 3 Biomolecular Interaction Centre, University of Canterbury, Private Bag 4800, Christchurch, 8140, New Zealand; 4 School of Biological Sciences, University of Canterbury, Private Bag 4800, Christchurch, 8140, New Zealand; 5 Electron Microscope Unit, University of Cape Town, Rondebosch, 7701, Cape Town, South Africa; 6 Computational Biology Group, Institute of Infectious Disease and Molecular Medicine, University of Cape Town, Observatory, 7925, Cape Town, South Africa; 7 Centre for High-Performance Computing, Rosebank, Cape Town, South Africa

## Abstract

**Background:**

*Maize streak virus *-strain A (MSV-A; Genus *Mastrevirus*, Family *Geminiviridae*), the maize-adapted strain of MSV that causes maize streak disease throughout sub-Saharan Africa, probably arose between 100 and 200 years ago via homologous recombination between two MSV strains adapted to wild grasses. MSV recombination experiments and analyses of natural MSV recombination patterns have revealed that this recombination event entailed the exchange of the movement protein - coat protein gene cassette, bounded by the two genomic regions most prone to recombination in mastrevirus genomes; the first surrounding the virion-strand origin of replication, and the second around the interface between the coat protein gene and the short intergenic region. Therefore, aside from the likely adaptive advantages presented by a modular exchange of this cassette, these specific breakpoints may have been largely predetermined by the underlying mechanisms of mastrevirus recombination. To investigate this hypothesis, we constructed artificial, low-fitness, reciprocal chimaeric MSV genomes using alternating genomic segments from two MSV strains; a grass-adapted MSV-B, and a maize-adapted MSV-A. Between them, each pair of reciprocal chimaeric genomes represented all of the genetic material required to reconstruct - via recombination - the highly maize-adapted MSV-A genotype, MSV-MatA. We then co-infected a selection of differentially MSV-resistant maize genotypes with pairs of reciprocal chimaeras to determine the efficiency with which recombination would give rise to high-fitness progeny genomes resembling MSV-MatA.

**Results:**

Recombinants resembling MSV-MatA invariably arose in all of our experiments. However, the accuracy and efficiency with which the MSV-MatA genotype was recovered across all replicates of each experiment depended on the MSV susceptibility of the maize genotypes used and the precise positions - in relation to known recombination hotspots - of the breakpoints required to re-create MSV-MatA. Although the MSV-sensitive maize genotype gave rise to the greatest variety of recombinants, the measured fitness of each of these recombinants correlated with their similarity to MSV-MatA.

**Conclusions:**

The mechanistic predispositions of different MSV genomic regions to recombination can strongly influence the accessibility of high-fitness MSV recombinants. The frequency with which the fittest recombinant MSV genomes arise also correlates directly with the escalating selection pressures imposed by increasingly MSV-resistant maize hosts.

## Background

Viruses are characteristically capable of rapid evolutionary adaptation. Typically, the primary driver of this adaptation is high basal mutation rates. For example, single-stranded RNA and DNA virus genomes generally accumulate 10^-4 ^to 10^-6 ^mutations per site per replication cycle [1-5]. Many viruses also experience high rates of homologous recombination and/or reassortment of genome components [6-10]. Acting either individually or in concert, these processes can create novel combinations of new and pre-existing genetic polymorphisms, generating substantial genetic diversity within a single, closely-related group of viruses (such as those within the same species [11,12]), or among viruses belonging to more distantly-related species, genera, or even families. In this way, new virus strains [13], species [14], genera [15,16], or - in at least some instances - families [17,18] can be formed. It is probably this capacity for rapid genetic diversification that has enabled the recent emergence of numerous economically and socially important pathogenic viruses of humans and their domesticated plants and animals.

One of these emergent pathogens is Maize streak virus strain A (MSV-A), which apparently arose around the mid 1800s via a recombination event between two *Digitaria sp*. adapted MSVs [3,13]. MSV-A is distributed throughout sub-Saharan Africa where it jeopardizes sustainable maize production in some of the world's poorest countries [19-21]. Its single component, circular, ~2.7 Kb, single-stranded DNA (ssDNA) genome encodes a movement protein (MP) and coat protein (CP) in the virion-sense [22-24], and in the complementary-sense the replication-associated proteins Rep and RepA [25-29]. Separating the virion- and complementary-sense open reading frames (ORFs) are the long intergenic region (LIR) - comprising transcriptional promoter elements and the virion strand origin of replication (*v-ori*) [30] - and a short intergenic region (SIR), where the transcription termination elements and the complementary strand origin of replication reside.

Experimental evolution can reveal key aspects of natural evolution, and has been used to study evolutionary processes leading to, for example, viral host-switching [31], resistance-breakage [32], and increased virulence [33]. With their small genome size, recombinogenic nature, and high mutation rates, geminiviruses have proved to be excellent models for experimental studies of the evolutionary mechanisms of virus emergence and adaptation. Accordingly, various experiments involving geminiviruses - and MSV in particular - have illuminated genetic factors underpinning important evolutionary processes, including the adaptation of these viruses to specific vector species [34] or hosts [35-37], their mutational dynamics [2,3,38-41], and the biochemical and selective factors constraining their adaptation through recombination [37,42-45].

We have previously described an experimental scheme for studying factors that affect the adaptive potential of recombination in mastrevirus evolution [45]. In this scheme, low-fitness laboratory-constructed reciprocal chimaeras of two wild-type (wt) MSV isolates (one naturally adapted to wild grasses, and the other adapted to maize) are co-introduced into a host plant, where they might re-create - via recombination - relatively high-fitness genomes that approximate the fittest wt genome. We used this experimental approach to identify maize-adaptive genetic polymorphisms within the MSV-A genome, and verified apparent hotspots of recombination detected in natural MSV populations at the v-*ori *and SIR [13,46], indicating that these regions of the genome are mechanistically predisposed to recombination. Moreover, this study demonstrated that a pair of co-infected low-fitness MSV genomes could efficiently recombine to regenerate genomes closely resembling wt MSV-A genotypes, and displaying fitness in maize that approached that of field-isolated MSV-A viruses.

In this previous study, only MSV-A-like genomes were recovered, suggesting that genomes representing positions of intermediate fitness within the sequence space were substantially less maize-adapted than the MSV-A-like genomes. This may have been due to the severe selective constraints of the MSV-resistant maize genotypes and/or to the specific pairs of chimaeric viruses used. Either way, these constraints limited the power and resolution of that specific experimental scheme. For example, the scheme provided no plausible way in which to recapitulate the evolutionary path that prototypical MSV-A genomes circulating in the mid 1800s may have taken - presumably via mutation and recombination, in a variety of differentially MSV-resistant maize plants - to traverse the maize-infecting fitness landscape towards the fitness peak occupied by extant MSV-A genotypes. It is plausible that such information may aid the elucidation of the actual history of MSV-A evolution, because other *in vitro *evolution studies have indicated that adaptive steps across fitness landscapes are constrained to relatively few, specific, evolutionary pathways [47-50].

Here we describe an improved version of the experimental system described by van der Walt *et al*. [45] that has enabled us to investigate the role played by host susceptibility, and the use of architecturally different pairs of defective starting chimaeras, in MSV evolution by recombination. In these experiments, we recovered recombinant genomes occupying a much wider variety of positions within the sequence space than those encountered in the original study, suggesting a plausible scenario for the initial adaptation of MSV to maize.

## Methods

### Viruses

Agro-infectious clones of wild-type MSV isolates MSV-VW [51] and MSV-MatA [52], as well as the laboratory-constructed reciprocal chimaeras of these viruses, MatMPCPVW and VWMPCPMat, and MatMPCPLIRVW and VWMPCPLIRMat [36] have been described previously. To explain the naming of the reciprocal chimaeras, the virus name following, say, the MP+CP segments in MatMPCPVW, indicates that the segments were derived from MSV-VW, whereas the rest of the MSV genome was derived from MSV-MatA (see Additional file 1).

### Agro-infection and leafhopper transmissions

We agro-inoculated 70 three-day-old seedlings of the MSV-sensitive maize genotype Sweetcorn cv. Golden Bantam (Millington Seed Co. USA), with a mixed inoculum of either MatMPCPVW+VWMPCPMat, or MatMPCPLIRVW+VWMPCPLIRMat as described by van der Walt *et al*. [45]. At approximately 30 days post inoculation (dpi) we transmitted viruses via leafhopper from each symptomatic plant to 13-day-old seedlings of the moderately MSV-resistant maize genotype PAN6099. For each transmission this was achieved by caging approximately eight *Cicadulina mbila *adults on a symptomatic Golden Bantam leaf for three days followed by transfer of cages to the third leaf of PAN6099 seedlings where they remained for the duration of the experiment [45,53]. We isolated DNA from symptomatic PAN6099 plants at approximately 30 dpi, and from symptomatic Golden Bantam plants at 60 dpi.

### Viral DNA isolation, cloning and sequencing

Viral DNA was isolated from symptomatic leaves using the Extract-n-Amp™Kit (Sigma-Aldrich), followed by rolling-circle amplification as previously described [54,55]. Amplified DNA was digested with the restriction enzyme *Bam*HI to generate ~ 2.7 kb monomeric MSV genomes which were gel-purified (GFX™, GE Healthcare), ligated into *Bam*HI-digested pGEM^®^-3Zf(+) (Promega Biotech) using T4 DNA ligase (Fermentas), and transformed into competent *Escherichia coli *(*E*. *cloni*^®^, Lucigen^® ^Corporation) using standard protocols [56]. The resulting positive clones were sequenced using universal M13 forward and reverse sequencing primers and previously-described internal primers [57]. The genome sequences of an additional 11 recombinant viruses from van der Walt *et al*. [45] were included in our analyses.

These, along with the viruses obtained in this study, were named using informative details such as, sequentially, the maize cultivar used (either Golden Bantam, [GB], or Pan6099, [Pan]), which recombination experiment they were obtained from (the MatMPCPVW+VWMPCPMat and MatMPCPLIRVW+VWMPCPLIRMat co-infections being, respectively, experiment 1 and 2), and following a hyphen, whether the viruses sequenced were either recombinants or parental input virus (labelled as R and I, respectively), and the specific plant number (and in a few cases followed also by the specific clone) from which the viruses were isolated.

### Construction of agro-infectious clones and fitness assays

Infectious clones of recombinant viruses were constructed in pBI121 (Clontech Laboratories, USA) as previously described [58]. The fitness of these cloned recombinants, along with that of wt viruses MSV-MatA and MSV-VW and each of the parental artificial chimaeras, was assessed in the moderately MSV-resistant maize genotype Sweetcorn cv. STAR 7714 (Starke Ayres, South Africa) by quantifying the percentage chlorotic leaf area produced by the viruses on leaves 4, 5 and 6 of symptomatically infected plants as previously described [59,60].

More specifically, with the exclusion of the virus GB1-R2, which was tested on approximately 42 separate plants, 18 of which became symptomatically infected, all the chlorotic areas caused by all of the viruses were assayed on leaves 4 through six for between 24 and 62 separate plants. Percentage chlorotic leaf areas caused by each virus on each plant were expressed as the mean (with 95% confidence interval) of the data obtained from leaves 4, 5 and 6.

### Statistical analysis

To test whether recombination breakpoints occurred more frequently in the coding or non-coding regions when using sensitive or resistant maize genotypes, we tallied the number of breakpoints occurring over the respective number of nucleotides (2219 nt for the coding regions, and 470 nt for the non-coding regions) and calculated a two-tailed p value using the Fisher's exact test.

Similarly, using the percentage pair-wise difference between recombinant viruses and MSV-MatA - obtained using the different pairs of parental viruses and maize genotypes - we calculated a two-tailed p value using a Mann-Whitney test to test for differences in the overall genomic similarity of recombinant viruses to MSV-MatA.

## Results and Discussion

### Recombination efficiently generates maize-adapted progeny from maladapted parental MSV genomes

We investigated the adaptive value of recombination during mixed MSV infections by tracing the trajectory of evolution via recombination across a sequence space bounded by maize-adapted and non-maize-adapted wt MSV genotypes. Specifically, we used a variation of a previously described experimental system [45] in which defective, laboratory-constructed MSV recombinants collectively comprising the complete genomic sequence of a maize-adapted MSV isolate were co-inoculated into maize and allowed to recombine during a defined time period. We assessed the efficiency with which maize adapted progeny genomes were generated within this simple experimental system, with respect to two important factors: (1) the particular partitioning of maize-adaptive genetic polymorphisms within defective parental viruses, and (2) the differential selective challenges imposed by different maize genotypes.

Seventy MSV-sensitive maize seedlings (Golden Bantam) were co-infected with each of the defective laboratory-constructed recombinant virus pairs MatMPCPVW+VWMPCPMat (containing reciprocal *mp *and *cp *exchanges between the maize-adapted MSV-A isolate, MSV-MatA, and the *Digitaria*-adapted MSV-B isolate, MSV-VW) and MatMPCPLIRVW+VWMPCPLIRMat (containing reciprocal *mp*, *cp *and LIR exchanges). From each set of agro-infections we identified approximately 60 plants with symptoms (chlorotic streaking and stunting) that ranged from mild to severe. At 60 dpi we isolated and sequenced a single MSV genome from each symptomatic plant. In addition, we transmitted viruses using leafhoppers from each of 36 symptomatic plants inoculated with MatMPCPLIRVW+VWMPCPLIRMat and five symptomatic plants inoculated with MatMPCPVW+VWMPCPMat to individual thirteen-day old MSV-resistant plants (PAN6099) as previously done [45].

We observed symptomatic infections in 15 MSV-resistant maize plants that were infected via leafhopper with viruses derived from the MatMPCPLIRVW+VWMPCPLIRMat co-infections, and in one symptomatic MSV-resistant maize plant infected with viruses obtained from the MatMPCPVW+VWMPCPMat co-infections. Approximately 60 days after the co-inoculations into Golden Bantam and 30 days after the leafhopper transmissions, we isolated and sequenced single MSV genomes from each infected MSV-resistant plant. We also analysed a further 11 MatMPCPVW+VWMPCPMat derived recombinants arising in MSV-resistant maize plants that were described by van der Walt *et al*. [45].

Complete genome sequences of the viruses isolated from symptomatic plants revealed that recombinant progeny arose frequently within the various mixed infections although this depended on the specific chimaeric nature of the co-inoculated viruses. While some of the viruses retrieved from MSV-sensitive maize were not recombinant, from the cohort of viruses transmitted via leafhoppers from sensitive to MSV-resistant maize all the isolated viruses were recombinant (Table 1; see also Additional file 2), presumably because of the greater selective pressures imposed by the resistant host, and/or due to a strong selective sieve during leafhopper transmission. As reported previously in co-inoculations with the MatMPCPVW+VWMPCPMat chimaera pair [45], we only retrieved parental viruses from some symptomatic plants. The parental viruses that were most frequently retrieved were those containing the maize adapted MSV-MatA derived *mp *and *cp *genes (i.e. VWMPCPLIRMat and VWMPCPMat). This was not surprising, since the *mp-cp *module of MSV-MatA has been shown to be the primary pathogenicity determinant of MSV in maize [36]. The additional contribution of the LIR to MSV pathogenicity [30,36] was also corroborated here by the fact that the parental virus, VWMPCPLIRMat, differing from VWMPCPMat only by the presence of the MSV-MatA LIR, was retrieved twice as often from symptomatic plants (Table 1).

**Table 1:** Recombinant genomes arising during mixed infections of different chimaeric parental MSV genomes in differentially MSV-resistant maize genotypes.

Input viruses	Maize genotype	Parental virus^b^		Recombinant virus^b^	Simple recombinants	Complex recombinants
MatMPCPVW + VWMPCPMat	MSV-sensitive	3% (1), MatMPCPVW	21% (7), VWMPCPMat	77% (26)	85%	15%

	MSV-resistant	0	0	100% (1)	75%	25%

MatMPCPLIRVW + VWMPCPLIRMat	MSV-sensitive	0	45% (18), VWMPCPLIRMat	55% (22)	95%	5%

	MSV-resistant	0	7% (1), VWMPCPLIRMat	93% (14)	79%	21%

MatMPCPVW + VWMPCPMat^a^	MSV-resistant	8% (1), MatMPCPVW	42% (5), VWMPCPMat	100% (11)	73%	27%

^a ^Experimental results obtained by van der Walt et al. [45]

^b^Numbers in brackets indicate the total number of individual plants from which MSV genomes were isolated

### Recombination breakpoint patterns are dependant on the starting parental chimaeras as well as the degree of host resistance

In agreement with our previous findings [45], the recombinant viruses recovered from these experiments displayed between two and 22 recombination breakpoints (i.e. they were a mixture of simple and complex recombinants), with higher proportions of simple recombinants being isolated from MSV-sensitive plants than from MSV-resistant plants (Table 1).

**Figure 1:**
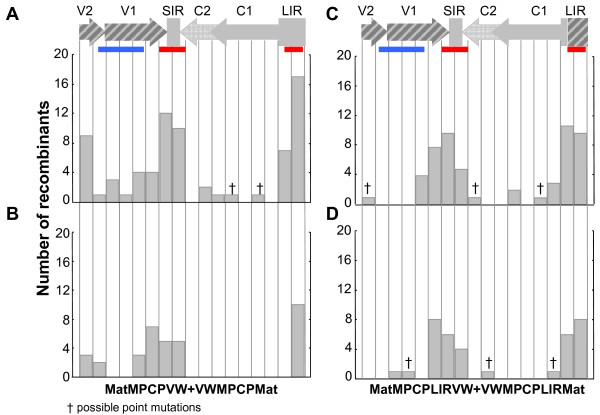
**Genome-wide distribution of recombination breakpoints arising during different recombination experiments**. A linearised MSV genome schematic divided into seventeen approximately equal segments. The light- and dark-grey hatched regions demarcate the regions swapped between the original wild-type viruses during construction of the chimaeric parental viruses (names indicated under each linearised genome). The total number of recombinants with breakpoints within each segment is represented using the bar graphs, where the recombinant viruses were obtained using the MatMPCPVW+VWMPCPMat chimaeric pair (panels A and B), or the MatMPCPLIRVW+VWMPCPLIRMat chimaeric pair (panels C and D) respectively in MSV-sensitive (panels A and C) and MSV-resistant (panels B and D) maize. The red and blue horizontal bars indicate, respectively, the approximate regions of prominent recombination hot-spots and cold-spots identified in wild-type MSV [13]. Genomic features: V2 = movement protein gene; V1 = coat protein gene; SIR = short intergenic region; C1/C2 = replication-associated protein gene; C1 = *rep*A gene; LIR = long intergenic region.

Although the two sets of laboratory-constructed parental chimaeras differed with respect to the partitioning of maize-adapted MSV genetic material between their constituent genomes, the recombination breakpoint distributions detected within the progeny recombinants mirrored those seen in natural geminiviruses [13,46,61-63]. Specifically, the majority of recombinant viruses had recombination breakpoints in previously identified mastrevirus and/or begomovirus recombination hot-spots such as at the *cp*/SIR interface and at the virion-strand origin of replication (*v*-*ori*) within the LIR [13,44,61,64,65] (Figure 1). However, contrary to natural breakpoint distributions observed in mastreviruses and begomoviruses, fewer recombination breakpoints fell in the complementary-sense genes, and more fell within the *cp *gene, particularly within the 3' half of the gene. Despite these differences between the natural and experimental recombination breakpoint distributions, both display a marked bias against recombination breakpoints within the protein-coding sequences, with the majority of cross-over events occurring in the intergenic regions (p-value < 0.0001 for each individual data set, or combined data set). It is likely that these coding region cold-spots are at least partially attributable to selection against the disruptive effects that recombination within genes can have on amino acid interactions within the tertiary and/or quaternary structures of recombinant proteins [66].

Notwithstanding the similarities between the recombination breakpoint distributions observed in the different experiments, there are two potentially important differences between recombinants arising during the MatMPCPVW+VWMPCPMat and MatMPCPLIRVW+VWMPCPLIRMat co-infections. While the recombination breakpoints in the 5' portion of *mp *have previously been observed in field-isolated MSV recombinants [13] and are not particularly unusual, in our experiments breakpoints were only observed within this region in co-infections initiated with the MatMPCPVW+VWMPCPMat chimaera pair. Martin and Rybicki [36] found genetic evidence of a possible *mp*-LIR interaction that might explain the selective advantage of recombination events in the 5' portion of *mp *that reunite LIR and *mp *sequences derived from the maize-adapted MSV-MatA isolate. Conversely, recombination breakpoints in this region during mixed infections of MatMPCPLIRVW and VWMPCPLIRMat would run the risk of separating MSV-MatA derived LIR and *mp *sequences, thereby possibly disrupting previously detected DNA-DNA or DNA-protein LIR - *mp *interactions (they have only been genetically detected and it is uncertain which of these interactions occur; [36]).

**Figure 2:**
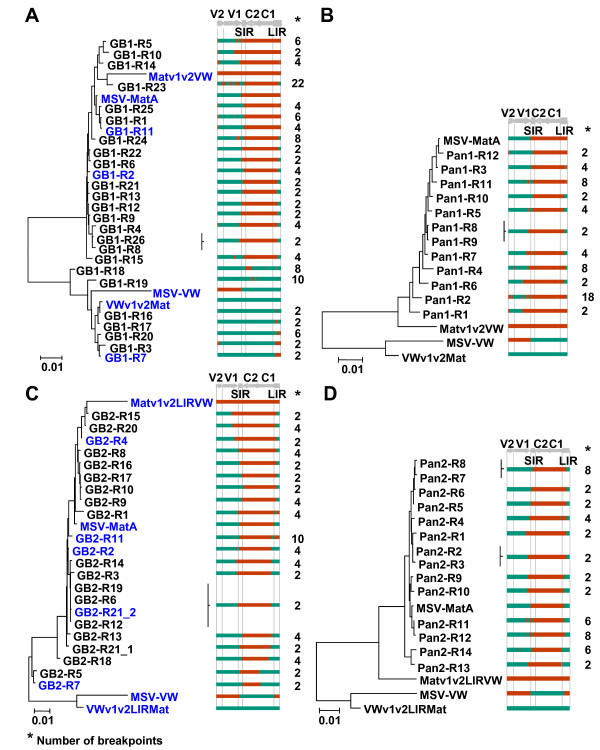
**Neighbour-joining tree depicting clustering of recombinant virus genotypes around that of MSV-MatA**. The recombinants arising during MatMPCPVW+VWMPCPMat or MatMPCPLIRVW+VWMPCPLIRMat chimaeric pair mixed infections in a MSV-sensitive maize genotype (panels A and C, respectively), and that either continue to persist or arise *de novo *following transmission of these viruses into a MSV-resistant maize genotype (panels B and D, respectively). The linearised genome schematics illustrate the recombination mosaics, where regions in orange are derived from MatMPCPVW or MatMPCPLIRVW viruses, and regions in green are derived from VWMPCPMat or VWMPCPLIRMat viruses. The panel on the right of each phylogenetic tree shows both the recombination pattern and number of breakpoints in each recombinant. The diagram above the mosaics shows the position of genomic features: V2 = movement protein gene; V1 = coat protein gene; SIR = short intergenic region; C1/C2 = replication-associated protein gene; C1 = *rep*A gene; LIR = long intergenic region. The viability of viruses highlighted in blue is shown in Figure 4.

A second observation worth noting is that the MSV-resistant and MSV-sensitive maize hosts gave rise to sets of recombinant viruses with different breakpoint distributions within the complementary-sense ORFs, C1 and C2. From the MSV-sensitive maize plants we identified six recombinants with breakpoints within this region (GB1-R18 and GB1-R19 in Figure 2A and GB2-R3, GB2-R5, GB2-R7, GB2-R18 and GB2-R21_1 in Figure 2C), while none were observed in this area of the recombinants isolated from the MSV-resistant maize. Two viruses obtained from resistant maize plants inoculated using leafhoppers previously fed on MatMPCPLIRVW+VWMPCPLIRMat co-infected plants, showed possible evidence of small recombination events in C1/C2 (Pan2-R4 and Pan2-R12, Figure 2D). However, these events would have only involved the exchange of a single polymorphic nucleotide and therefore could not be reliably distinguished from convergent point mutations. Although recombination within the C1/C2 genes of field-isolated MSVs has been observed, the parental viruses shared greater than ~95% sequence identity in all of these cases [13]. The presence of recombination breakpoints within this region in viruses that we isolated from sensitive maize plants implies that there is no biochemical impediment to recombination breakpoints falling in C1/C2 when parental viruses are <95% identical (in all cases here the parental viruses were 89% identical). Rather, the absence of breakpoints in this region in the resistant maize plants strongly implies that such recombinants are possibly usually defective, and that natural selection is responsible for their apparent rarity in nature.

Most of the recombinants produced during our experiments were essentially reconstructions of the original maize-adapted wt parental virus, MSV-MatA, with one breakpoint occurring within 200 nucleotides (nt) upstream or downstream of the *v-ori*, and another close to the *cp*-SIR interface (Figure 2). Importantly, and consistent with previous findings that the *v*-*ori *is a recombination hotspot [13,37,44,64], 42% of all MatMPCPVW+VWMPCPMat recombinants (10/26 from the MSV-sensitive maize and 6/12 from the MSV-resistant maize) analysed in this study as well as 64% (7/11 from the MSV-resistant maize) analysed previously by van der Walt *et al*. [45], and 47% of MatMPCPLIRVW+VWMPCPLIRMat derived recombinants (9/22 from MSV-sensitive maize and 8/14 from MSV-resistant maize) had a breakpoint within 14 nt of this site (Figure 2).

Besides transfers of extensive contiguous sequences comprising nearly entire virion-sense or complementary-sense gene cassettes, many signals of transfers of much smaller fragments were also observed. While some of these may have represented the exchange of a single polymorphic nucleotide, it is difficult to distinguish such recombination events from possible convergent point mutations, and we therefore did not consider them any further with respect to our recombination analysis. Small recombination events entailing the exchange of two or more polymorphic nucleotides were not apparently clustered and occurred throughout the genome in the LIR (recombinants GB1-R23, Pan1-R11 and Pan1-R3), *cp *(recombinants GB1-R1 and Pan1-R11), *mp *(recombinants GB1-R23, GB2-R18 and Pan1-R2) and C1/C2 (recombinants GB1-18 and GB1-R19; see Figure 2A and 2B).

Although almost all of the recombinants were unique to the plants from which they were isolated, occasionally identical recombinants were isolated from different plants suggesting that certain "recombinant solutions" were more easily accessible and/or selectively favoured. Examples of such recombinants derived from the MatMPCPVW+VWMPCPMat co-infections include GB1-R8 and GB1-R26 isolated from MSV-sensitive maize plants (Figure 2A), and Pan1-R8 and Pan1-R9 isolated from MSV-resistant maize plants (Figure 2B). Examples from the MatMPCPLIRVW+VWMPCPLIRMat co-infections include GB2-R6, GB2-R19, GB2-R12 and GB2-R21_2 isolated from MSV-sensitive maize (Figure 2C), and Pan2-R7 and Pan2-R8 or Pan2-R2 and Pan2-R3 (Figure 2C) isolated from MSV-resistant maize plants. Conversely, we also identified instances, exemplified by GB2-R21_1 and GB2-R21_2 (Figure 2C), where two different recombinants were isolated from the same plant.

### Recombinants tend to converge on the MSV-MatA genotype

Given the potential importance of genetic recombination during evolutionary adaptation, we were interested in assessing the efficiency with which recombination could reassemble a genome resembling MSV-MatA - the easily accessible, and presumably "optimal", maize-adapted "target solution" genome that we used to construct the reciprocal parental chimaeras. In our experiments, recombination enabled the exploration of vast tracts of sequence space bounded by, in one dimension, the parental genomes used during the co-infections and, in a second dimension, the original wt viruses MSV-MatA and MSV-VW used to construct these parental genomes.

The simplest way in which all 248 MSV-MatA-derived polymorphisms carried by a set of reciprocal chimaeras could have recombined to form a single progeny genome was via two crossover events, at the junctions used to construct the parental chimaeric genomes. While the majority of the recombinant genomes appeared to approximately represent such simple cross-over events, the degree to which progeny genomes recovered MSV-MatA-derived polymorphisms varied according to the particular pair of reciprocal chimaeric viruses in the experiment, and with the type of host plant.

In recombinants recovered from MatMPCPVW+VWMPCPMat co-infections of the sensitive maize genotype, 31.5% - 97.3% of polymorphic sites were identical to MSV-MatA. In contrast, recombinant progeny from the same reciprocal chimaeric parental genome pair isolated from resistant maize carried 88.3% - 98.2% of the MSV-MatA polymorphisms.

The recombinants arising from MatMPCPLIRVW+VWMPCPLIRMat co-infections were generally more closely related to MSV-MatA than were recombinants isolated from MatMPCPVW+VWMPCPMat co-infections, with MSV-MatA contributing between 71.4% - 97.3% of polymorphisms in recombinants isolated from MSV-sensitive maize, and between 92.9% - 99.2% of polymorphisms in recombinants isolated from resistant maize.

**Figure 3:**
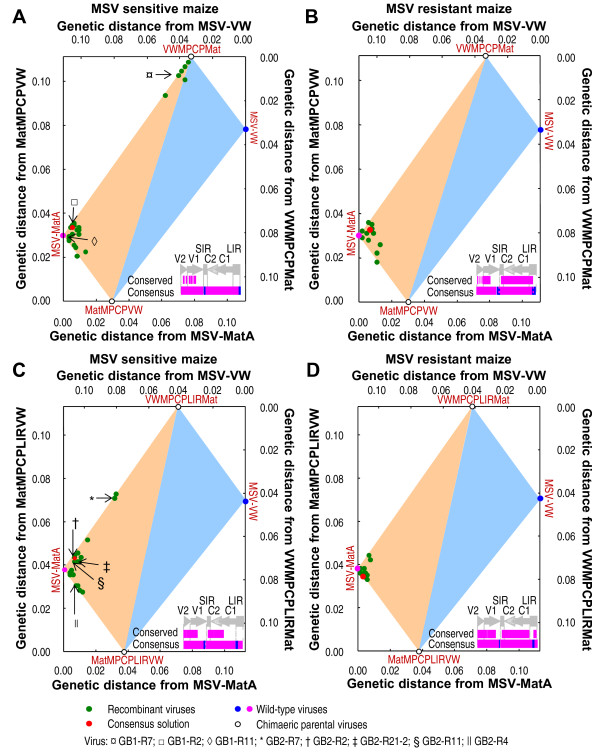
**Distribution of recombinant viruses arising in maize within a two dimentional projection of the sequence space bounded by wild-type and chimaeric parental viruses**. A Hamming distance graph showing the effects of maize genotype and chimaeric parental sequence pairs on how precisely the recombinant genotypes that arise during mixed infections converge on the target solution, MSV-MatA. Each green circle represents a single recombinant virus obtained using the MatMPCPVW+VWMPCPMat chimaeric pair in (A) MSV-sensitive and (B) MSV-resistant maize, and MatMPCPLIRVW+VWMPCPLIRMat chimaeric pair in (C) MSV-sensitive and (D) MSV-resistant maize. The red circle in each graph indicates the position of the consensus solution. The genome schematic embedded within each graph shows the regions in pink and blue derived from, respectively, MSV-MatA and MSV-VW. The conserved regions and overall consensus solutions are shown. The genome regions are as indicated in the legend of Figure 1. The viability of the viruses indicated using the symbols ¤ (GB1-R7), (GB1-R2), ◊ (GB1-R11), * (GB2-R7), † (GB2-R2), ‡ (GB2-R21-2), § (GB2-R11), || (GB2-R4) is shown in Figure 4.

The differences in the extent to which recombinants in the four separate experiments converged on the MSV-MatA sequence is reflected in the conserved and consensus sequence polymorphism maps in Figure 3. Importantly, irrespective of the specific experimental conditions, only MSV-A-derived polymorphisms were absolutely conserved amongst all the recombinants obtained from each of the different experiments (see the conserved solution maps in Figure 3 indicating the origins of the invariant sites across all observed recombinants). Moreover, the consensus sequence of the recombinants (that is, the genome constructed from the most common polymorphisms observed at each variable site across all the observed recombinants) was between 93.69% and 96.87% similar to the MSV-MatA sequence in all four of the experiments.

Whereas the recombinants isolated from MatMPCPVW+VWMPCPMat co-infections of MSV-sensitive maize shared only a few conserved polymorphic sites around the 3' end of *mp *and the 5' portion of *cp *(Figure 3A), conserved sites amongst the recombinants from the MSV-resistant maize genotype additionally included all sites within C1/C2 and many sites within the LIR and SIR regions (Figure 3B). In both host genotypes, the ''conserved'' regions of recombinants from MatMPCPLIRVW+VWMPCPLIRMat co-infections included sites in the 3' portion of *mp*, the 5' portion of *cp*, most of the C2 and the C2 proximal half of the SIR (Figure 3C and 3D). Recombinants obtained from resistant maize additionally contained MSV-A derived polymorphisms throughout most of their C1 and C2 ORFs and within the V2 proximal portion of their LIR sequences immediately downstream of the *v-ori *(Figure 3D).

Although the "consensus" recombinant progeny genomes arising from the different parental chimaera pairs in the two hosts were all remarkably MSV-MatA-like, some MSV-VW derived polymorphisms were invariably present around the sites used in the initial construction of the chimaeras from MSV-MatA and MSV-VW (Figure 3). Whereas in MatMPCPVW+VWMPCPMat derived recombinants the MSV-VW polymorphisms within the consensus occurred downstream of the *v-ori*, in the MatMPCPLIRVW+VWMPCPLIRMat derived recombinants they occurred upstream of this site. Although this pattern doubtlessly reflects the differences between the ligation sites used to construct the two reciprocal chimaeric pairs, it also indicates that the *v-ori *is a recombination hotspot.

### MSV-sensitive maize hosts provide a more permissive fitness landscape

Although the ''consensus'' recombinant genomes generated under the different experimental conditions were very similar, there were notable differences in the relative ease with which recombination between the different co-infected parental chimaera pairs yielded recombinants that approximated MSV-MatA. For example, although the recombinants obtained from MSV-sensitive plants inoculated with MatMPCPVW+VWMPCPMat showed the most diverse recombination patterns, these recombinants were collectively not significantly less similar to MSV-MatA than those obtained from MSV-resistant plants, which displayed much less diverse patterns of recombination (p = 0.57, Mann-Whitney U-test; Figure 3A and 3B; see Additional file 3). However, recombinant viruses isolated from MSV-sensitive plants co-infected with MatMPCPLIRVW+VWMPCPLIRMat were significantly less like MSV-MatA than those obtained from MSV-resistant plants (p = 0.0014, Mann-Whitney U-test; Figure 3C and 3D; see Additional file 3).

In the MSV-sensitive host, the recombinant viruses produced by each pair of chimaeric parental viruses were indistinguishable with respect to their similarity to MSV-MatA (p = 0.66, Mann-Whitney U-test; see Additional file 3). In contrast, in MSV-resistant maize, the recombinants derived from MatMPCPLIRVW+VWMPCPLIRMat co-infections were collectively much more similar to MSV-MatA than their MatMPCPVW+VWMPCPMat-derived counterparts (p = 0.0015, Mann-Whitney U-test; see Additional file 3).

These results suggest that host susceptibility, as well as the configuration of parental genomes, can influence the efficiency with which fit genomes are assembled via recombination in a mixed infection. In other words, not only does a more selective maize host limit the possible trajectories of MSV evolution by reducing the diversity of arising recombinants, but it also is more selective of maize-adapted polymorphisms.

However, it remains unlikely that different degrees to which recombinants converge on the MSV-MatA sequence in co-infections of different parental chimaera pairs are attributable to selection alone. Rather, we expected that the parental chimaera pair that was assembled using cloning sites closest to the biochemically predisposed recombination hotspots, either within the LIR near the *v-ori *or within the SIR, would converge more easily on the ideal MSV-A solution. The reason for this is that recombination events at these sites would be most likely to reverse the steps used to construct the original parental chimaeras and to yield the wt maize-adapted genome (in this case MSV-MatA). This expectation was borne out by the observation that the cloning sites used to construct the MatMPCPLIRVW+VWMPCPLIRMat chimaera pair (the pair yielding recombinants that converged most closely on the MSV-MatA target solution) were on average 45 nt closer to the biochemically predisposed recombination hotspots than those used to construct the MatMPCPVW+VWMPCPMat chimaera pair.

### The fitness of recombinant genomes

The recombinant genomes that we recovered occupied a variety of positions within the sequence space separating MSV-MatA from MSV-VW (see Figure 3A and 3C). We hypothesised that MSV-MatA occupies a peak within the fitness landscape, and we therefore compared the fitness in maize of eight of the recombinants recovered from our experiments with that of MSV-MatA, MSV-VW and their parental reciprocal chimaeras. As an approximate measure of viral fitness, we agroinoculated a moderately MSV-resistant maize genotype (Sweetcorn cv. STAR 7714) and quantified the percentage chlorotic leaf areas on leaves 4, 5 and 6 of successfully infected plants. The selection of this genotype was due to it being amenable to producing more discriminative infection data, than either the MSV-sensitive genotype within which most of the tested viruses produced indistinguishable symptoms, or the MSV-resistant genotype within which some of the viruses produced no symptoms at all. Although increased pathogenicity does not necessarily equate with increased fitness in nature, in the context of MSV infecting individual maize plants, replicative fitness and pathogenicity seem to be quite highly correlated (43, 52).

**Figure 4:**
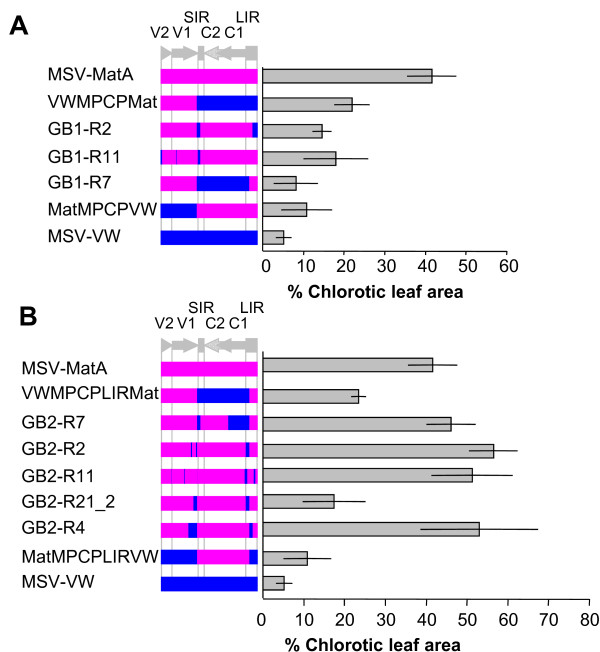
**Fitness assay of parental MSV genomes and recombinant MSV viruses**. The viability of recombinant viruses compared to that of their chimaeric parents, and the wild-type viruses MSV-MatA and MSV-VW. Recombinants recovered from MSV-sensitive plants (cv. Golden Bantam) after agroinoculation with the pair MatMPCPVW+VWMPCPMat (A) and MatMPCPLIRVW+VWMPCPLIRMat (B) were tested in moderately-resistant maize (cv. STAR 7714). Mean chlorotic leaf areas (and the 95% confidence intervals of these estimates indicated by error bars) observed on leaves 4, 5 and 6 of symptomatic plants. Shown in blue and pink on the genome schematic are regions derived from MSV-VW and MSV-MatA, respectively. The genome regions are as indicated in the legend of Figure 1.

All of the recombinants from MatMPCPVW+VWMPCPMat co-infections, namely GB1-R2, GB1-R7 and GB1-R11, produced symptoms that were less severe than those of VWMPCPMat, the more virulent of the two parental chimaeras. One recombinant, GB1-R7, was even less severe than MatMPCPVW - the least virulent of the two parental chimaeras - but was nevertheless slightly more severe than MSV-VW (Figure 4A). A comparison of the mean percentage chlorotic leaf area produced by the three recombinants suggests that symptom severity correlated with genetic distance from the MSV-MatA genomic sequence. Thus, of the three recombinants, GB1-R7 was the least like MSV-A (only 36.9% of the 248 MSV-MatA/MSV-VW polymorphic nucleotides were derived from MSV-MatA), and was also the least virulent. GB1-R2, which derived 94.6% of its polymorphic nucleotides from MSV-A, was slightly more virulent, and GB1-R11 was the most genetically similar to MSV-A (97.3% of the polymorphic nucleotides are from MSV-MatA) and produced the most severe symptoms. While these data suggest an apparent trend, one should note both that the 95% confidence intervals (CI) of the mean symptom severity estimates of these recombinants overlap extensively (Figure 4A) and that the correlation between genetic distance from MSV-MatA and symptom severity is not statistically supported (p = 0.43; Spearman ranks test).

Of the five recombinants derived from MatMPCPLIRVW+VWMPCPLIRMat co-infections, four (GB2-R2, GB2-R4, GB2-R7, and GB2-R11) produced symptoms as severe as MSV-MatA, but one (GB2-R21_2) was only slightly more severe than MSV-VW and MatMPCPLIRVW (Figure 4B). GB2-R7 was the least MSV-A-like of these recombinants (72.3% of the polymorphic sites were derived from MSV-MatA) and accordingly produced less severe symptoms than did the more MSV-MatA-like recombinants GB2-R4, GB2-R11, and GB2-R2 (which respectively obtained 93.8%, 94.6%, and 94.6% of their polymorphic sites from MSV-MatA). However, the 95% CI of the chlorotic leaf area measurements made for these viruses were all largely overlapping (Figure 4B) and they probably do not have very different degrees of pathogenicity in maize. It is noteworthy that in addition to being clustered closer to the ideal solution, MSV-MatA, the MatMPCPLIRVW+VWMPCPLIRMat derived recombinants were also on average substantially fitter than those derived using the MatMPCPVW+VWMPCPMat chimaera pair (Figure 4A and 4B; p = 0.064 Mann Whitney U-test).

Although GB2-R21_2 was more MSV-A-like than GB2-R7 (respectively 92.9% and 72.3% of polymorphic sites derived from MSV-MatA), the symptoms it produced in maize were significantly less severe than those produced by all of the other recombinants tested. It is noteworthy however, that GB2-R21_2 carries a mutation in the C1 ORF that differentiates it from both MSV-VW and MSV-MatA. Although this mutation is silent for both Rep and RepA, it could conceivably have an adverse effect on ssDNA genomic or transcribed RNA secondary structures, as has been demonstrated for other silent Rep mutations [67,68].

Although of low resolution, our survey of the fitness landscape surrounding the maize-adapted MSV-MatA genotype and the non-maize-adapted MSV-VW genotype in the maize cultivar Sweetcorn (STAR 7714) suggests a feasible evolutionary trajectory leading to the maize-adapted MSV-A strain (represented here by MSV-MatA[52]). Being mildly symptomatic in MSV-sensitive maize genotypes [60,61,65,69], the *Digitaria*-adapted MSV-B (represented here by MSV-VW), -G and -F strains probably occupy the 'lowlands' of the "MSV in maize" fitness landscape. In addition to natural examples [13], our experiments demonstrate that recombination between these strains resulting in the exchange of a maize-specific pathogenicity determinant, the *mp*-*cp *gene module [36], could have greatly improved the fitness of the ancestral MSV-A virus. Even in our most permissive maize genotype, large portions of the maize-adapted MSV-MatA-derived *mp*-*cp *sequence were conserved across all the recombinants. From this point onwards, as is implied by the relative fitness value of all the recombinants we tested, our results suggest that further exploration of the fitness landscape in permissive maize genotypes, either by further recombination or by point mutation, could have enabled a prototypical recombinant MSV-A to progressively climb higher on the fitness landscape to eventually attain the altitudes that have been reached by MSV-A genotypes found today throughout Africa [70].

## Conclusions

Using an established model system for analysing the evolution and adaptation of MSV to maize plants, we demonstrate that despite diverse recombinants emerging during mixed infections involving two separate sets of parental viruses and two different hosts, both the over-all distributions of recombination breakpoints and the average patterns of recombination are remarkably similar across all experimental conditions. Most notably, in all experiments the consensus of all observed recombinants deterministically converged upon that of the maize-adapted MSV-A genotype, MSV-MatA, which was initially used to construct the parental chimaera pairs. Besides converging on the MSV-MatA sequence, when tested in isolation some of the recombinants also produced symptoms in maize that approached those produced by MSV-MatA.

It is clear from our study that the biochemically predisposed recombination hot-spots within the MSV genome strongly influenced the recombination patterns that we observed in our experiments. It is also evident that varying mechanistic predispositions to recombination across the MSV genome can constrain the efficiency with which recombination provides access to fitness peaks within the sequence space. However, it is noteworthy that we have provided evidence in MSV that, as is becoming apparent in other viruses such as Human immunodeficiency virus [71], recombination hotspots within the intergenic regions correspond with genome sites at which recombination breakpoints are likely to have the smallest deleterious impact on virus viability - a finding which suggests that the MSV genome may have specifically evolved to accommodate a recombinogenic life-style.

Finally, our results indicate a plausible scenario for the creation and early evolution of the maize adapted MSV-A strain through recombination between two *Digitaria*-adapted MSVs. The complex recombination patterns that we have sometimes observed indicate that within a permissive MSV-sensitive maize genotype, the prototypical MSV-A genome could conceivably have been assembled through a series of adaptive recombination events (and possibly also compensatory and/or adaptive point mutations) that incrementally nudged it towards the fitness peak that MSV-A currently populates in maize.

## Authors' contributions

ALM, EvdW, DPM, EPR conceived the study and participated in its design. ALM, DPM, AV isolated, cloned and sequenced the viral genomes and performed basic viral bioinformatics. ALM, DPM carried out plant inoculations and virulence assays, analyzed and interpreted the data. ALM, DPM, EvdW, AV, EPR drafted and revised the manuscript. All authors read and approved the final manuscript.

## Supplementary Material

Additional file 1 **Genome organization of wild-type and chimaeric MSV genomes used in this study**. The curved arrows indicate open reading frames (ORFs) diverging from the long intergenic region (LIR) and eventually converging on the short intergenic region (SIR). The intergenic regions, the ORFs in the complementary-sense - which encode the replication-associated protein (Rep) and the replication-associated protein (RepA) - and the ORFs in the virion-sense - which encode the movement protein (MP) and the coat protein (CP) - are colored red (in the case of MSV-VW) or blue (in the case of MSV-MatA). This color-code is also used to delineate the genomic portions of MSV-MatA and MSV-VW used to construct the pair of reciprocal chimaeric MSV genomes used to conduct recombination experiments. Click here for file

Additional file 2 **Alignment of wild-type and recombinant MSV genomes**. Full genome sequence alignment of MSV viruses aligned from the start codon of the movement protein gene. The wild-type viruses MSV-MatA and MSV-VW, as well as the chimaeric parental viruses and recombinants used in two different recombination experiments are included. The file is in FASTA format and should be viewed using a sequence analysis program such as Mega5. Click here for file

Additional file 3 **Pair-wise distance of recombinant viruses from MSV-MatA**. Distribution of recombinant viruses using percentage pair-wise distance and statistical analysis (Mann-Whitney U test, two-tailed p value) of the approximation of each group of viruses to MSV-MatA. Recombinant viruses were obtained using different pairs of parental chimaeric MSV genomes, inoculated into differentially-resistant maize genotypes. Click here for file
