# Supplementary material for: Recombination hotspots and host susceptibility modulate the adaptive value of recombination during maize streak virus evolution
Source: BMC Evol Biol. 2011 Dec 2;11:350. doi: 10.1186/1471-2148-11-350 (PMC3280948; doi:10.1186/1471-2148-11-350)
Supplement: Additional file 1 — Genome organization of wild-type and chimaeric MSV genomes used in this study. The curved arrows indicate open reading frames (ORFs) diverging from the long intergenic region (LIR) and eventually converging on the short intergenic region (SIR). The intergenic regions, the ORFs in the complementary-sense - which encode the replication-associated protein (Rep) and the replication-associated protein (RepA) - and the ORFs in the virion-sense - which encode the movement protein (MP) and the coat protein (CP) - are colored red (in the case of MSV-VW) or blue (in the case of MSV-MatA). This color-code is also used to delineate the genomic portions of MSV-MatA and MSV-VW used to construct the pair of reciprocal chimaeric MSV genomes used to conduct recombination experiments. [file 1471-2148-11-350-S1.PPT]

## Slide 1
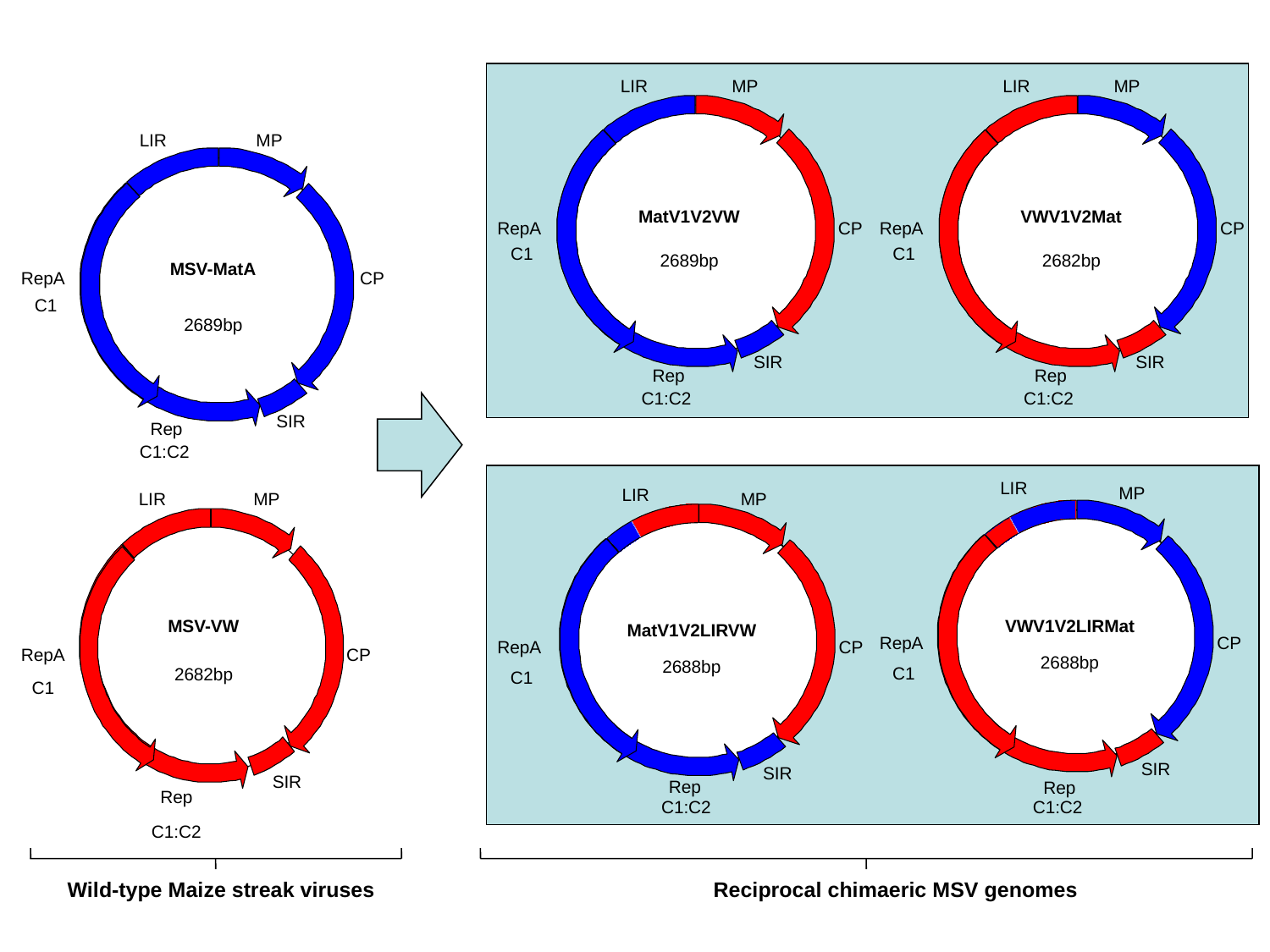

LIR
MP
MatV1V2VW
RepA
CP
C1
2689bp
SIR
Rep
C1:C2
LIR
MP
VWV1V2Mat
RepA
CP
C1
2682bp
SIR
Rep
C1:C2
LIR
MP
MSV-MatA
RepA
CP
C1
2689bp
SIR
Rep
C1:C2
LIR
MP
VWV1V2LIRMat
RepA
CP
2688bp
C1
SIR
Rep
C1:C2
LIR
MP
MatV1V2LIRVW
RepA
CP
2688bp
C1
SIR
Rep
C1:C2
LIR
MP
MSV-VW
RepA
CP
2682bp
C1
SIR
Rep
C1:C2
Wild-type Maize streak viruses
Reciprocal chimaeric MSV genomes
