# Supplementary material for: Recombination hotspots and host susceptibility modulate the adaptive value of recombination during maize streak virus evolution
Source: BMC Evol Biol. 2011 Dec 2;11:350. doi: 10.1186/1471-2148-11-350 (PMC3280948; doi:10.1186/1471-2148-11-350)
Supplement: Additional file 3 — Pair-wise distance of recombinant viruses from MSV-MatA. Distribution of recombinant viruses using percentage pair-wise distance and statistical analysis (Mann-Whitney U test, two-tailed p value) of the approximation of each group of viruses to MSV-MatA. Recombinant viruses were obtained using different pairs of parental chimaeric MSV genomes, inoculated into differentially-resistant maize genotypes. [file 1471-2148-11-350-S3.PPT]

## Slide 1
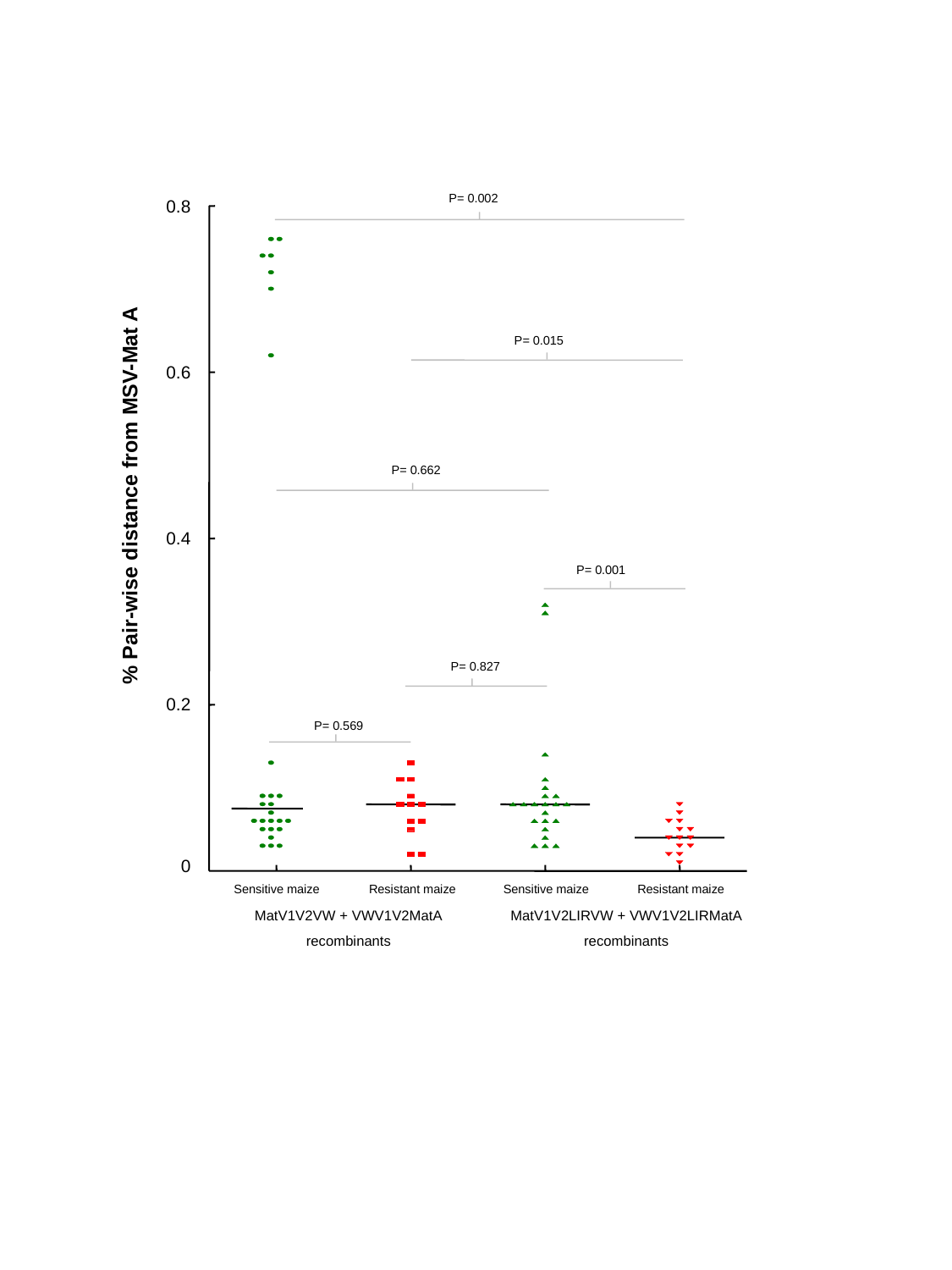

P= 0.002
0.8
P= 0.015
0.6
P= 0.662
% Pair-wise distance from MSV-Mat A
0.4
P= 0.001
P= 0.827
0.2
P= 0.569
0
Sensitive maize
Resistant maize
Sensitive maize
Resistant maize
MatV1V2VW + VWV1V2MatA
recombinants
MatV1V2LIRVW + VWV1V2LIRMatA
recombinants
